# Mobile phones, cordless phones and rates of brain tumors in different age groups in the Swedish National Inpatient Register and the Swedish Cancer Register during 1998-2015

**DOI:** 10.1371/journal.pone.0185461

**Published:** 2017-10-04

**Authors:** Lennart Hardell, Michael Carlberg

**Affiliations:** Department of Oncology, Faculty of Medicine and Health, Örebro University, Örebro, Sweden; Universidad de Navarra, SPAIN

## Abstract

We used the Swedish Inpatient Register (IPR) to analyze rates of brain tumors of unknown type (D43) during 1998–2015. Average Annual Percentage Change (AAPC) per 100,000 increased with +2.06%, 95% confidence interval (CI) +1.27, +2.86% in both genders combined. A joinpoint was found in 2007 with Annual Percentage Change (APC) 1998–2007 of +0.16%, 95% CI -0.94, +1.28%, and 2007–2015 of +4.24%, 95% CI +2.87, +5.63%. Highest AAPC was found in the age group 20–39 years. In the Swedish Cancer Register the age-standardized incidence rate per 100,000 increased for brain tumors, ICD-code 193.0, during 1998–2015 with AAPC in men +0.49%, 95% CI +0.05, +0.94%, and in women +0.33%, 95% CI -0.29, +0.45%. The cases with brain tumor of unknown type lack morphological examination. Brain tumor diagnosis was based on cytology/histopathology in 83% for men and in 87% for women in 1980. This frequency increased to 90% in men and 88% in women in 2015. During the same time period CT and MRI imaging techniques were introduced and morphology is not always necessary for diagnosis. If all brain tumors based on clinical diagnosis with CT or MRI had been reported to the Cancer Register the frequency of diagnoses based on cytology/histology would have decreased in the register. The results indicate underreporting of brain tumor cases to the Cancer Register. The real incidence would be higher. Thus, incidence trends based on the Cancer Register should be used with caution. Use of wireless phones should be considered in relation to the change of incidence rates.

## Introduction

During use of mobile phones and cordless phones the brain is the main target organ for radiofrequency (RF) radiation emitted from these devices [1,2]. Thus, an increased risk for brain tumors has been of concern for a long time. This initiated an evaluation of the scientific evidence on the risk by the International Agency for Research on Cancer (IARC) at the World Health Organization (WHO) in May 2011. An increased risk for glioma and acoustic neuroma was concluded based on human epidemiological studies. RF radiation in the frequency range 30 kHz–300 GHz, was evaluated to be a Group 2B, i.e. a ‘possible’, human carcinogen [3,4].

The use of handheld wireless phones (mobile and cordless phones) and brain tumor risk is still regarded to be controversial in spite of results from different research group showing an increased risk. We evaluated glioma risk using the Bradford Hill viewpoints from 1965 on association or causation [5]. We concluded that all nine viewpoints seemed to be fulfilled. This conclusion was corroborated in a recent meta-analysis [6]. Thus, RF radiation should be regarded to cause glioma. Similarly an increased risk for acoustic neuroma has been reported in several investigations; see Hardell et al [7].

It has been suggested that results from descriptive studies on brain tumor incidence may be used to contradict analytical epidemiological studies on brain tumor risk associated with use of wireless phones [8]. However descriptive epidemiology without any individual exposure data is less reliable than studies that have assessed use of wireless phones on an individual basis. Furthermore register data may have several shortcomings just as are inherent in the Swedish Cancer Register. We have made a thorough discussion of that issue in a previous publication [9].

Register studies on brain tumor incidence should be focused on type of tumor, i.e. glioma and localization in the brain, especially the temporal lobe. In England increasing incidence of glioblastoma multiforme (astrocytoma grade IV), especially in the frontal and temporal lobes, has been found for the time period 2003–2013, under publication, see also Carlberg, Hardell [5]. A real increase in the incidence of glioblastoma multiforme in the frontal and temporal lobes and cerebellum was reported in USA [10]. However, not all cancer registers provide data on anatomical localization of the tumor.

In an ecological study from England annual incidence of brain tumors in the temporal and parietal lobes were modeled based on population-level covariates. The study period was 1985–2014. Malignant brain tumors in the temporal lobe increased faster than would be expected. Using a latency period of 10 years this increase was related to the penetration of mobile phone use. This corresponded to an additional increase of 35% (95% Credible Interval 9%; 59%) or 188 (95% CI 48–324) additional cases annually [11]. The author concluded that the findings were in agreement with mobile phones and other wireless equipment being causing factors.

We published recently increasing rates of brain tumors of unknown type (ICD-10 code D43) in Sweden based on the National Inpatient Register (IPR) and Causes of Death Register (CDR) during the time period 1998–2013 [9]. We used joinpoint regression analysis of the trends in number of patients per 100,000 in IPR. The average annual percentage change in IPR was statistically significant yielding +1.78%, 95% confidence interval (CI) +0.76, +2.81%, in men and women combined. The change was somewhat higher in men (+2.03%, 95% CI +0.52, +3.56%) than in women (+1.58%, 95% CI +0.77, +2.40%).

### Aim of the study

The aim of this study was to further analyze trends of brain tumors of unknown type (D43) in IPR adding two more years (2014–2015) and to study the trend in different age groups. Furthermore, one additional aim was to study the incidence of brain tumors in the Swedish Cancer Register. We wanted also to study glioma risk associated with use of mobile phones or cordless phones in different age groups at the time of diagnosis in our previous case-control study [12].

### Ethics

There were two parts of the study. The register based part with no individual data such as name and personal identification number. This part did not need approval by Ethics Committee. All information is freely available in open data bases. The case-control studies were approved by the Ethics Committee (Örebro County Hospital DNR 351/96, Uppsala University DNR 2005:367). Physicians provided written consent to include the respective patient in the study. Informed written consent was obtained by the cases when answering the questionnaire. In addition they gave written consent to obtain copies of X-rays and histopathological investigations. All communication was by postal questionnaires and letters. This consent procedure was approved by the ethics committees.

## Material and methods

### Study design

Rates of brain tumors of unknown type, D43, were studied using IPR without any personal identification information: (http://www.socialstyrelsen.se/statistik/statistikdatabas/diagnoserislutenvard).

It was established in 1964 and has complete national coverage since 1987 [13]. Register data on D43 are available from 1998. Currently more than 99% of hospital discharges are registered. Data were analysed for the time period 1998–2015. We report number of patients per 100,000 inhabitants. Age-standardized rates are not available in the register.

The Swedish Cancer Register is administered by the National Board of Health and Welfare (http://www.socialstyrelsen.se/statistik/statistikdatabas/cancer). We analysed incidence per 100,000 person-years age-adjusted according to the World population for the time period 1998–2015. This was the same time period as for IPR so as to be able to compare the results. We selected ICD-7 code 193.0 = brain tumor and also histopathological codes 475 = astrocytoma grades I, II and 476 = astrocytoma grades III, IV. The register gives data for each gender but not for men and women in total.

The second part of this study concerned our case-control studies on brain tumor cases diagnosed during 1997–2003 or 2007–2009. Population based matched controls were used and they were ascertained from the Swedish Population Register. Detailed information on materials and methods has been published by us previously [12]. All cases had histopathological verified brain tumor. Both men and women were included aged 20–80 years (1997–2003) and 18–75 years (2007–2009) at the time of diagnosis. Only living subjects were included. Tumor localisation in the brain was based on reports to the cancer registries and medical records. Only cases with glioma (n = 1,380) were included; astrocytoma grade I, II (n = 221), astrocytoma grade III, IV (n = 857), oligodendroglioma (n = 162), other/mixed glioma (n = 140).

Exposures were assessed using a mailed questionnaire sent to each person. Regarding use of a mobile phone, the time of average use (min per day) was estimated, as well as first year of use and number of years. Use of cordless desktop phones was covered by similar questions; years, average daily use, use of a hands-free device, and preferred ear. Use of the wireless phone was referred to as ipsilateral (≥50% of the time) or contralateral (<50% of the time) in relation to tumor side, for more details see Hardell, Carlberg [12].

### Statistical methods

The Joinpoint Regression Analysis program, version 4.1.1.1 was used to examine number of patients per 100,000 in inpatient care and incidence per 100,000 person-years in the Swedish Cancer Register, by fitting a model of 0–3 joinpoints using permutation tests with Bonferroni correction for multiple testing to calculate the number of joinpoints that best fits the material [14]. When joinpoints were detected annual percentage changes (APC) and 95% confidence intervals (CIs) were calculated for each linear segment. Average annual percentage changes (AAPC) were also calculated for the whole time period using the average of the APCs weighted by the length of the segment. To be able to calculate APC and AAPC the data was log-transformed prior to analysis. Thus, it was not possible to perform joinpoint regression analysis when there were years with no cases during the time period.

StataSE 12.1 (Stata/SE 12.1 for Windows; StataCorp., College Station TX) was used for the analyses of the case-control studies. Odds ratios (OR) and 95% confidence intervals (CI) were calculated using unconditional logistic regression including the whole control sample (i.e. matched to both malignant and benign cases) to increase the power of the study. Use in a car with external antenna and/or use of a hands-free device were disregarded. A minimum latency period of ≤1 year of exposure was used for exposure. Adjustment was made for gender, age (as a continuous variable), year of diagnosis and socioeconomic index (SEI) divided into four categories (blue-collar worker, white-collar worker, self-employed, unemployed).

## Results

### National Inpatient Register (IPR)

#### D43 tumor of unknown type in the brain or CNS

AAPC increased statistically significant in men for all ages during 1998–2015 with +2.19%, 95% CI +1.04, +3.36%, Table 1.

**Table 1:** Trends in number of patients per 100,000 with ICD-10 code D43 in the Swedish National Inpatient Register (IPR) during 1998–2015 for different age groups estimated by joinpoint regression analysis. APC = Annual Percentage Change; AAPC = Average Annual Percentage Change. For detected joinpoints, years for locations are indicated and trends are given for each time period (APC 1 = time from 1998 to first joinpoint; APC 2 = time from first to second joinpoint; APC 3 = time from second joinpoint to 2015). Empty cells indicate no joinpoints detected.

Age group	Joinpoint location	APC 1 (95% CI)	APC 2 (95% CI)	APC 3 (95% CI)	AAPC (95% CI)
**Men**					
All ages (n = 8,013)	2007	+0.25 (-1.37, +1.88)	+4.42 (+2.42, +6.46)		+2.19 (+1.04, +3.36)
0–19 years (n = 509)	No joinpoint detected				+1.39 (-1.44, +4.30)
20–39 years (n = 834)	No joinpoint detected				+2.71 (+0.91, +4.55)
40–59 years (n = 2,193)	No joinpoint detected				+1.69 (+0.60, +2.79)
60–79 years (n = 3,644)	No joinpoint detected				+1.43 (+0.52, +2.35)
80+ years (n = 833)	No joinpoint detected				+0.94 (-0.85, +2.75)
**Women**					
All ages (n = 7,237)	2008	+0.27 (-0.68, +1.23)	+4.54 (+2.84, +6.27)		+2.01 (+1.20, +2.82)
0–19 years (n = 465)	No joinpoint detected				+2.24 (+0.87, +3.62)
20–39 years (n = 682)	No joinpoint detected				+2.70 (+0.67, +4.77)
40–59 years (n = 1,902)	No joinpoint detected				+1.09 (-0.38, +2.57)
60–79 years (n = 3,180)	2005	-1.01 (-4.09, +2.17)	+3.36 (+1.48, +5.29)		+1.54 (-0.01, +3.11)
80+ years (n = 1,008)	2001, 2009	+13.69 (-5.61, +36.94)	-4.05 (-8.70, +0.84)	+7.23 (+0.69, +14.19)	+2.82 (-1.26, +7.07)
**Total**					
All ages (n = 15,250)	2007	+0.16 (-0.94, +1.28)	+4.24 (+2.87, +5.63)		+2.06 (+1.27, +2.86)
0–19 years (n = 974)	No joinpoint detected				+1.77 (-0.08, +3.66)
20–39 years (n = 1,516)	No joinpoint detected				+2.71 (+1.08, +4.37)
40–59 years (n = 4,095)	No joinpoint detected				+1.40 (+0.32, +2.49)
60–79 years (n = 6,824)	No joinpoint detected				+1.60 (+0.80, +2.40)
80+ years (n = 1,841)	No joinpoint detected				+0.97 (-0.52, +2.49)

**Fig 1:**
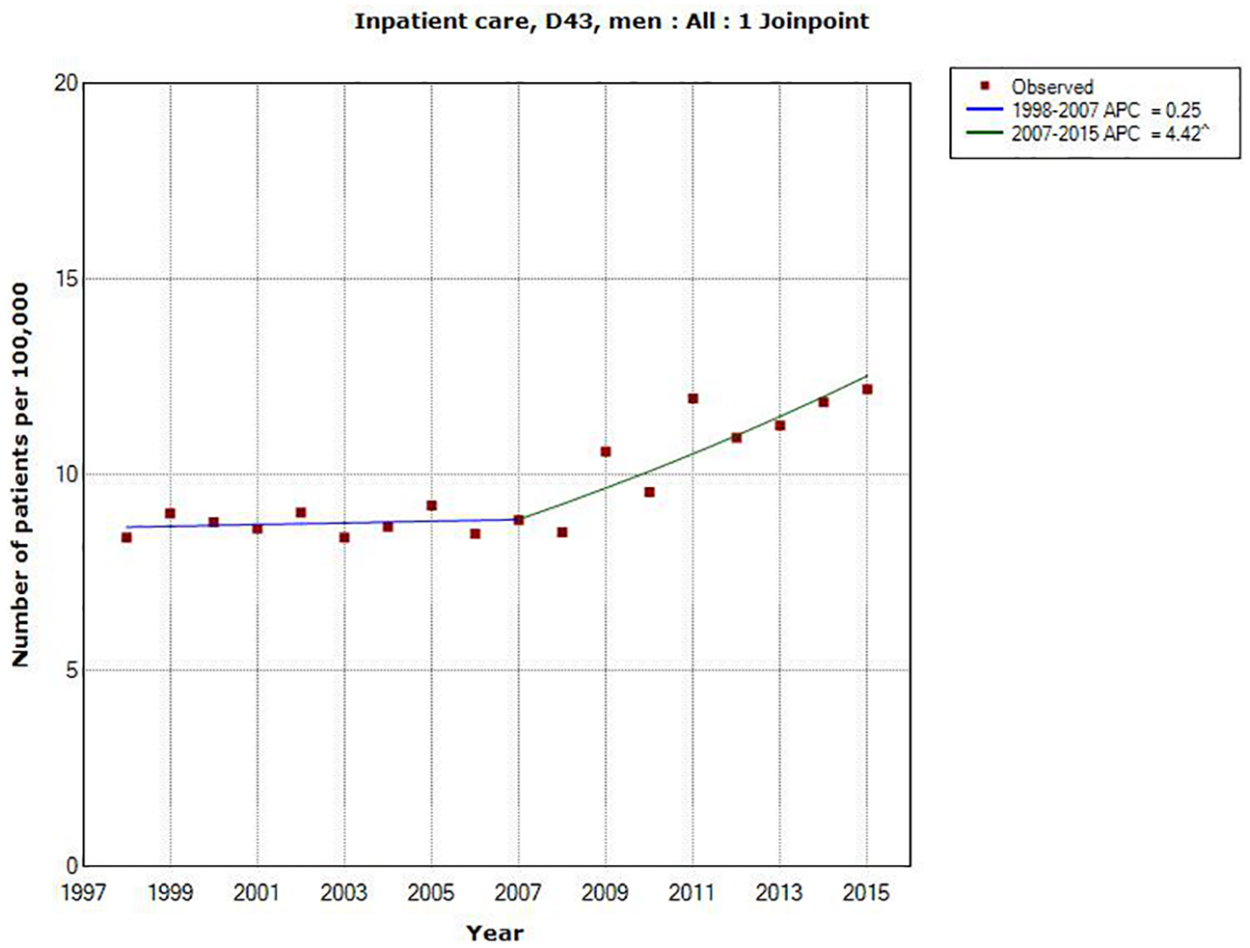
Joinpoint regression analysis of number of patients per 100,000 inhabitants according to the Swedish National Inpatient Register for men, all ages during 1998–2015 diagnosed with D43 = tumor of unknown type in the brain or CNS. (http://www.socialstyrelsen.se/statistik/statistikdatabas/diagnoserislutenvard).

A joinpoint was detected in 2007 in men (1998–2007: APC +0.25%, 95% CI -1.37, +1.88%; 2007–2015: APC +4.42%, 95% CI +2.42, +6.46%), see also Fig 1.

**Fig 2:**
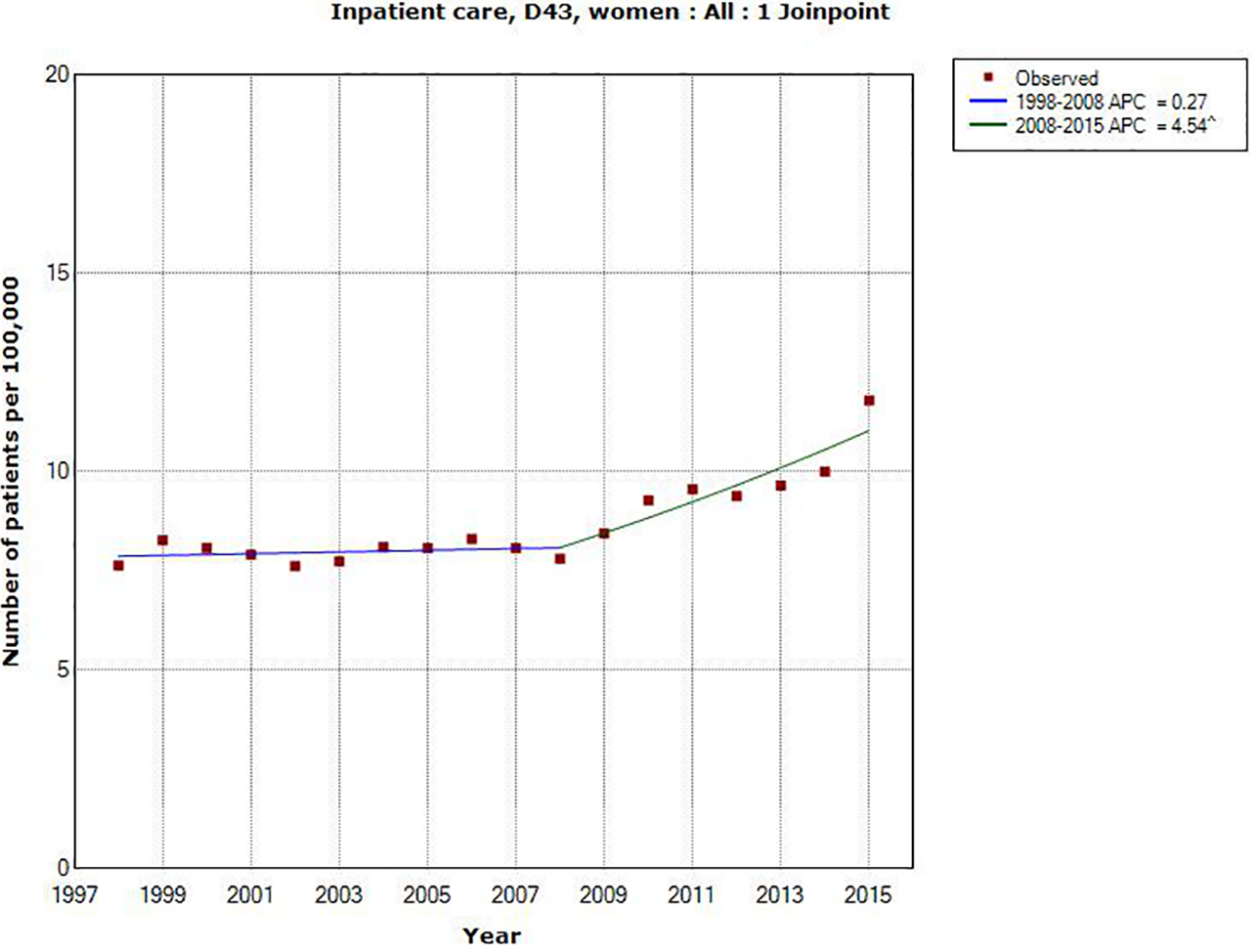
Joinpoint regression analysis of number of patients per 100,000 inhabitants according to the Swedish National Inpatient Register for women, all ages during 1998–2015 diagnosed with D43 = tumor of unknown type in the brain or CNS. (http://www.socialstyrelsen.se/statistik/statistikdatabas/diagnoserislutenvard).

Also in women AAPC increased statistically significant during 1998–2015, AAPC +2.01%, 95% CI +1.20, +2.82%. A joinpoint was detected in 2008 (1998–2008: APC +0.27%, 95% CI -0.68, +1.23%; 2008–2015: APC +4.54%, 95% CI +2.84, +6.27%), Table 1, Fig 2.

**Fig 3:**
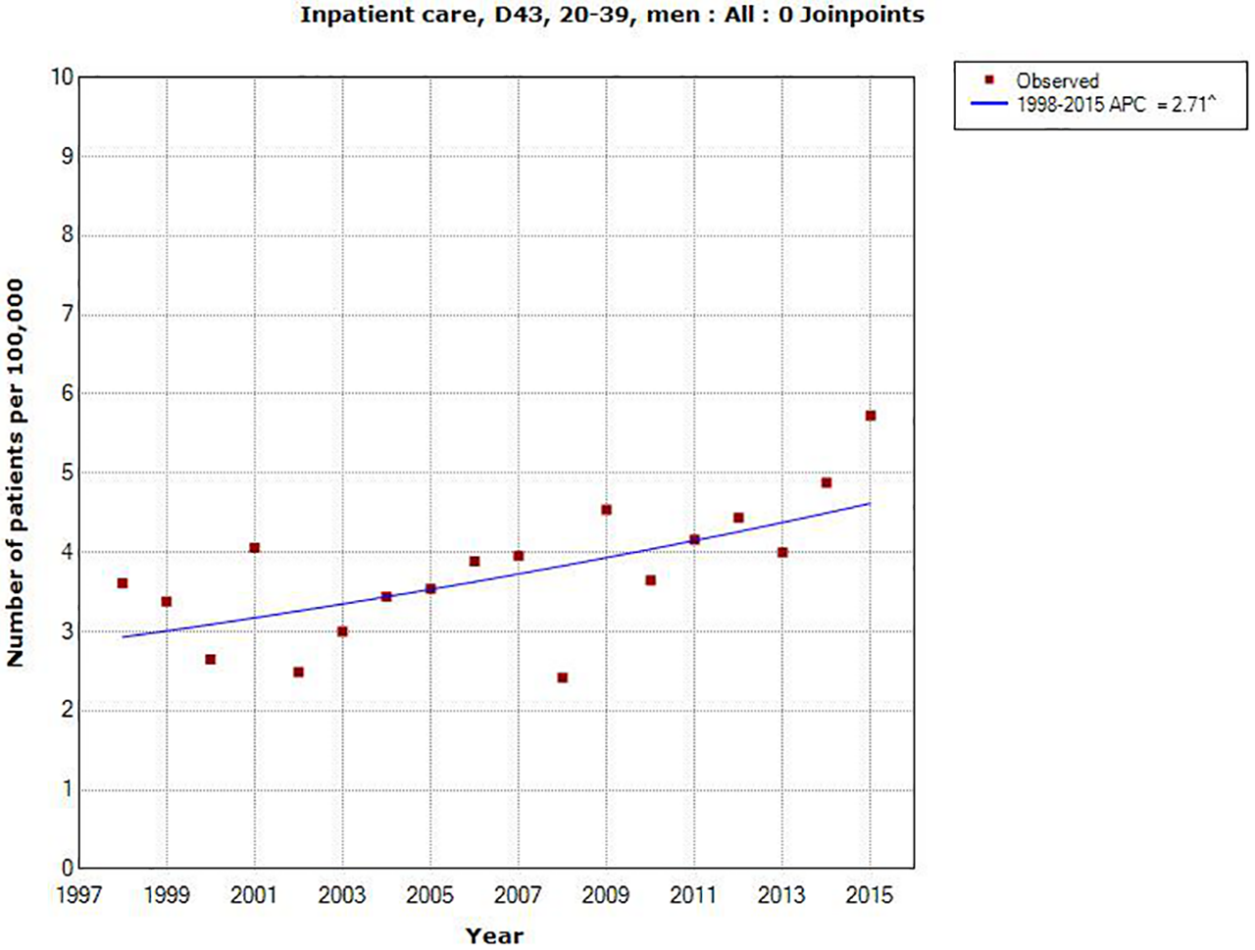
Joinpoint regression analysis of number of patients per 100,000 inhabitants according to the Swedish National Inpatient Register for men aged 20–39 years during 1998–2015 diagnosed with D43 = tumor of unknown type in the brain or CNS. (http://www.socialstyrelsen.se/statistik/statistikdatabas/diagnoserislutenvard).

**Fig 4:**
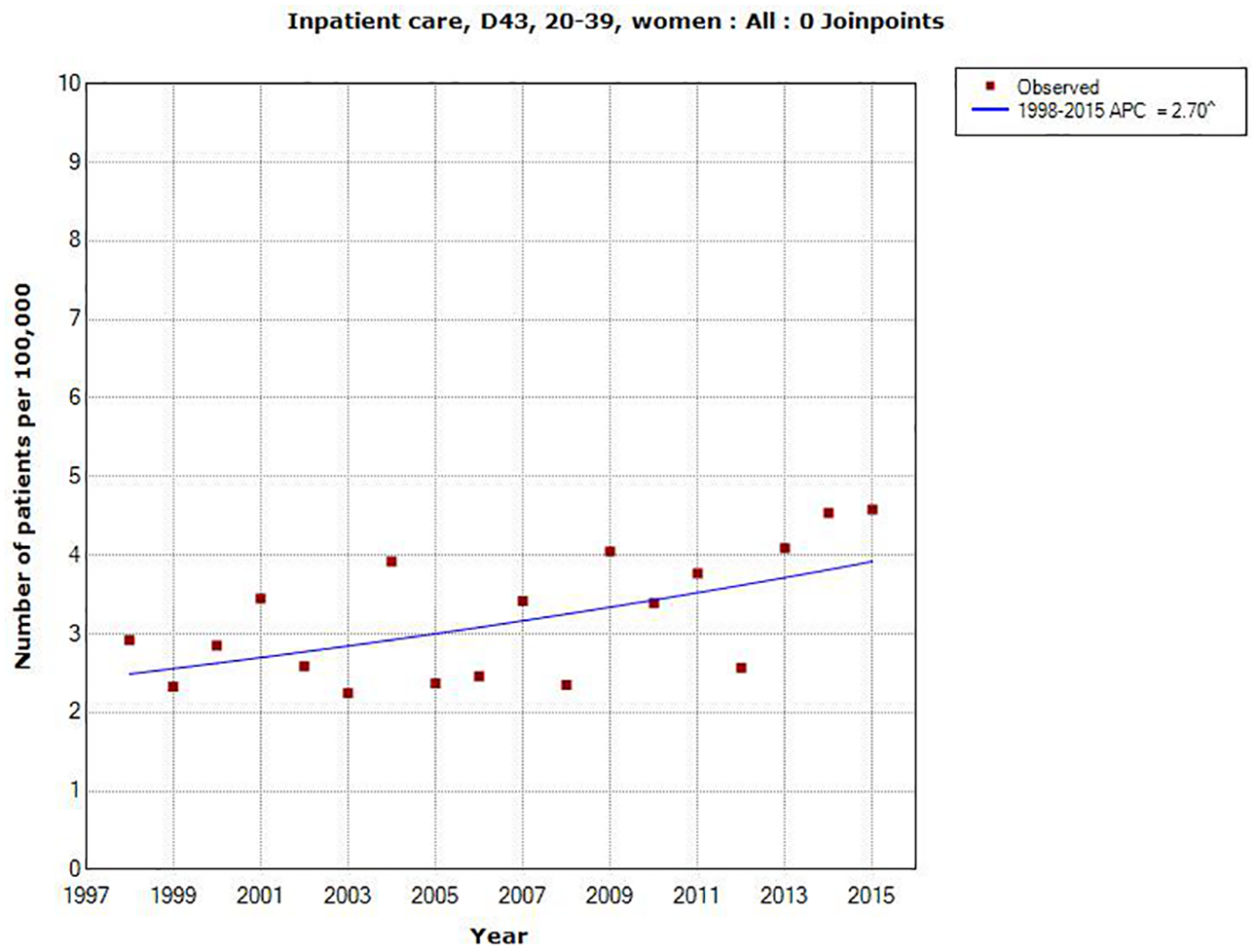
Joinpoint regression analysis of number of patients per 100,000 inhabitants according to the Swedish National Inpatient Register for women aged 20–39 years during 1998–2015 diagnosed with D43 = tumor of unknown type in the brain or CNS. (http://www.socialstyrelsen.se/statistik/statistikdatabas/diagnoserislutenvard).

Also in different age groups AAPC increased during the time period 1998–2015 although not statistically significantly so in men aged 0–19 and 80+ years and in women aged 40+ years, Table 1. Highest increase in total (both genders) was found for 20–39 years; AAPC +2.71%, 95% CI +1.08, +4.37%; in men AAPC +2.71%, 95% CI +0.91, +4.55% and in women AAPC +2.70%, 95% CI +0.67, 4.77%, see Figs 3 and 4.

One joinpoint was detected for women aged 60–79 and two joinpoints in the age group 80+, see Table 1.

### The Swedish Cancer Register

#### ICD-7 code 193.0 = brain tumor including brain, meninges and CNS nerves

In men AAPC increased statistically significant during 1998–2015 with +0.49%, 95% CI +0.05, +0.94%, see Table 2. Also in women AAPC increased although not statistically significant, +0.33%, 95% CI -0.29, +0.95%.

**Table 2:** Trends in age-standardized incidence rates per 100,000 in men and women in the Swedish Cancer Register during 1998–2015 estimated by joinpoint regression analysis. AAPC = Average Annual Percentage Change.

Age group	AAPC % Brain tumor ICD-7 code 193.0 (95% CI)	AAPC % Astrocytoma grade I, II (95% CI)	AAPC % Astrocytoma grade III, IV (95% CI)
**Men**			
All ages	+0.49 (+0.05, +0.94) (n = 10,248)	-0.13 (-2.49, +2.18)* (n = 1,227)	+0.81 (-0.002, +1.62) (n = 4,412)
0–19 years	+0.37 (-1.24, +1.99) (n = 790)	+0.27 (-2.02, +2.62) (n = 297)	-**(n = 58)
20–39 years	+0.72 (-0.39, +1.86) (n = 1,326)	+0.59 (-1.68, +2.91) (n = 369)	+0.20 (-2.10, +2.56) (n = 364)
40–59 years	-0.36 (-1.07, +0.36) (n = 3,319)	+0.88 (-0.97, +2.76) (n = 366)	-0.26 (-0.99, +0.48) (n = 1,621)
60–79 years	+0.30 (-2.57, +3.25)*** (n = 4,257)	+0.34 (-3.49, +4.32) (n = 183)	+1.68 (+0.39, +2.99) (n = 2,275)
80+ years	+2.21 (+0.32, +4.15) (n = 556)	-** (n = 12)	** (n = 94)
**Women**			
All ages	+0.33 (-0.29, +0.95) (n = 11,445)	+0.30 (-1.37, +1.99) (n = 1,018)	+0.16 (-0.68, +1.01) (n = 2,947)
0–19 years	-0.96 (-2.34, +0.45) (n = 679)	-0.73 (-3.09, +1.68) (n = 265)	-0.27 (-3.45, +3.01) (n = 64)
20–39 years	+0.82 (-0.12, +1.76) (n = 1,227)	+0.47 (-2.33, +3.35) (n = 290)	-0.27 (-5.70, +5.41) (n = 219)
40–59 years	+0.03 (-0.85, +0.93) (n = 4,049)	+1.43 (-1.04, +3.96) (n = 310)	-1.12 (-2.71, +0.50) (n = 988)
60–79 years	+1.09 (+0.08, +2.12) (n = 4,744)	+3.08 (-1.19, +7.53) (n = 144)	+1.38 (+0.32, +2.45) (n = 1,585)
80+ years	+1.65 (-0.23, +3.56) (n = 746)	-** (n = 9)	+3.37 (-2.63, +9.73) (n = 91)

*One joinpoint detected; Annual Percentage Change (APC) 1 1998–2002–6.27, 95% CI -15.06, +3.44; APC 2 2002–2015 +1.83%, 95% CI +0.18, +3.51%.

** Not possible to perform joinpoint regression analysis since no cases were found in certain years.

***Two joinpoints detected; APC 1 1998–2001–8.72%, 95% CI -15.88, -0.96; APC 2 2001–2004 +11.49, 95% CI -5.30, +31.25; APC 3 2004–2015–0.02%, 95% CI -1.11, +1.09.

Somewhat higher increase of AAPC than in all ages combined was found in the age group 20–39 years; in men +0.72%, 95% CI -0.39, +1.86%, and in women +0.82%, 95% CI -0.12, +1.76%. AAPC increased in all age groups except for men 40–59 years and women 0–19 years. Statistically significant increase was found only in men 80+, AAPC +2.21%, 95% CI +0.32, +4.15% and in women aged 60–79 years, AAPC +1.09, 95% CI +0.08, +2.12%, Table 2.

#### Astrocytoma

For men with astrocytoma grade I, II one joinpoint was detected in 2002 (APC 1998–2002: -6.27%, 95% CI -15.06, +3.44%; APC 2002–2015: +1.83%, 95% CI +0.18, +3.51%). AAPC increased in all age groups although not statistically significant. No joinpoint was detected in different age groups, Table 2. Also in women AAPC increased although not statistically significant.

**Fig 5:**
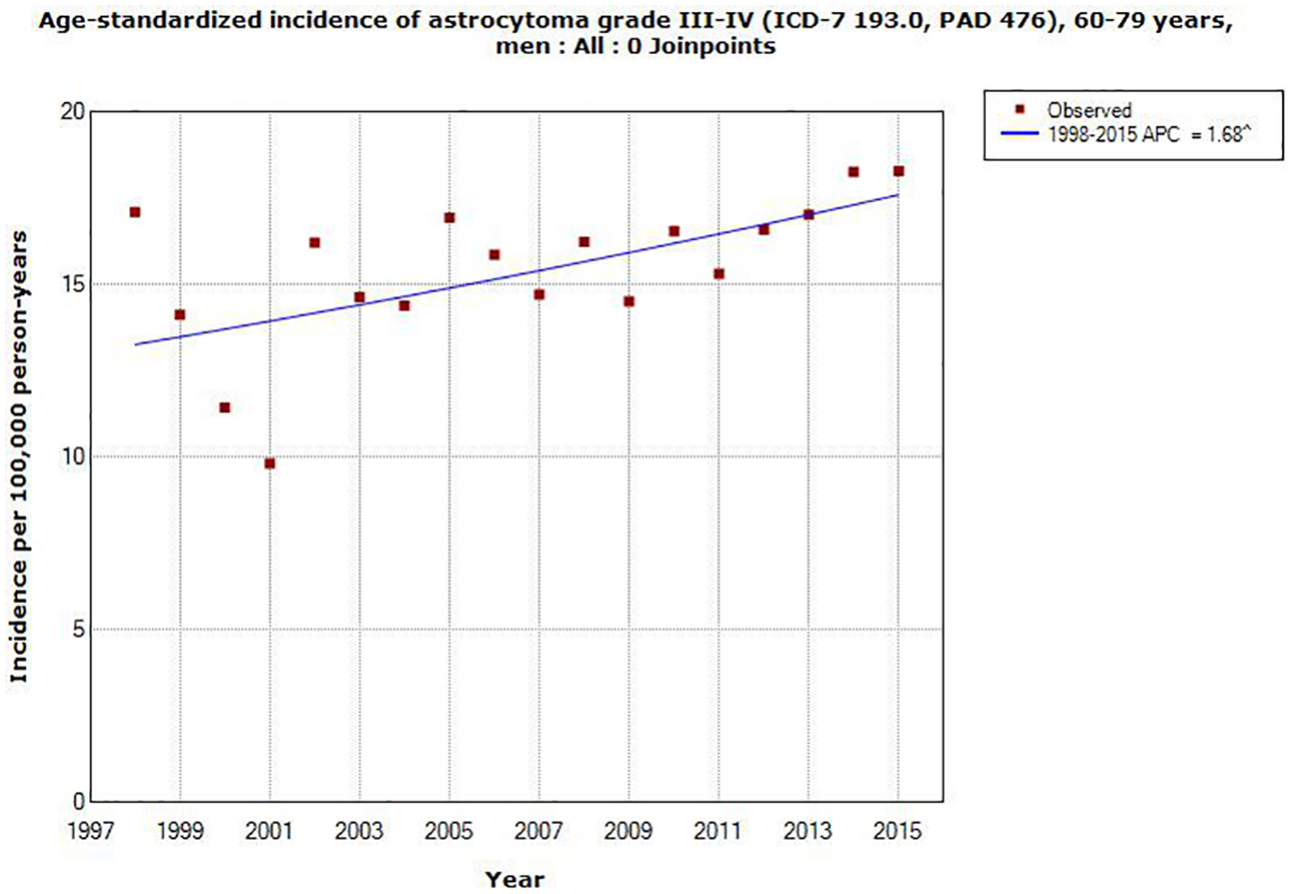
Joinpoint regression analysis of age-standardized incidence rates per 100,000 in men aged 60–79 years with astrocytoma grade III or IV in the Swedish Cancer Register during 1998–2015. (http://www.socialstyrelsen.se/statistik/statistikdatabas/cancer).

**Fig 6:**
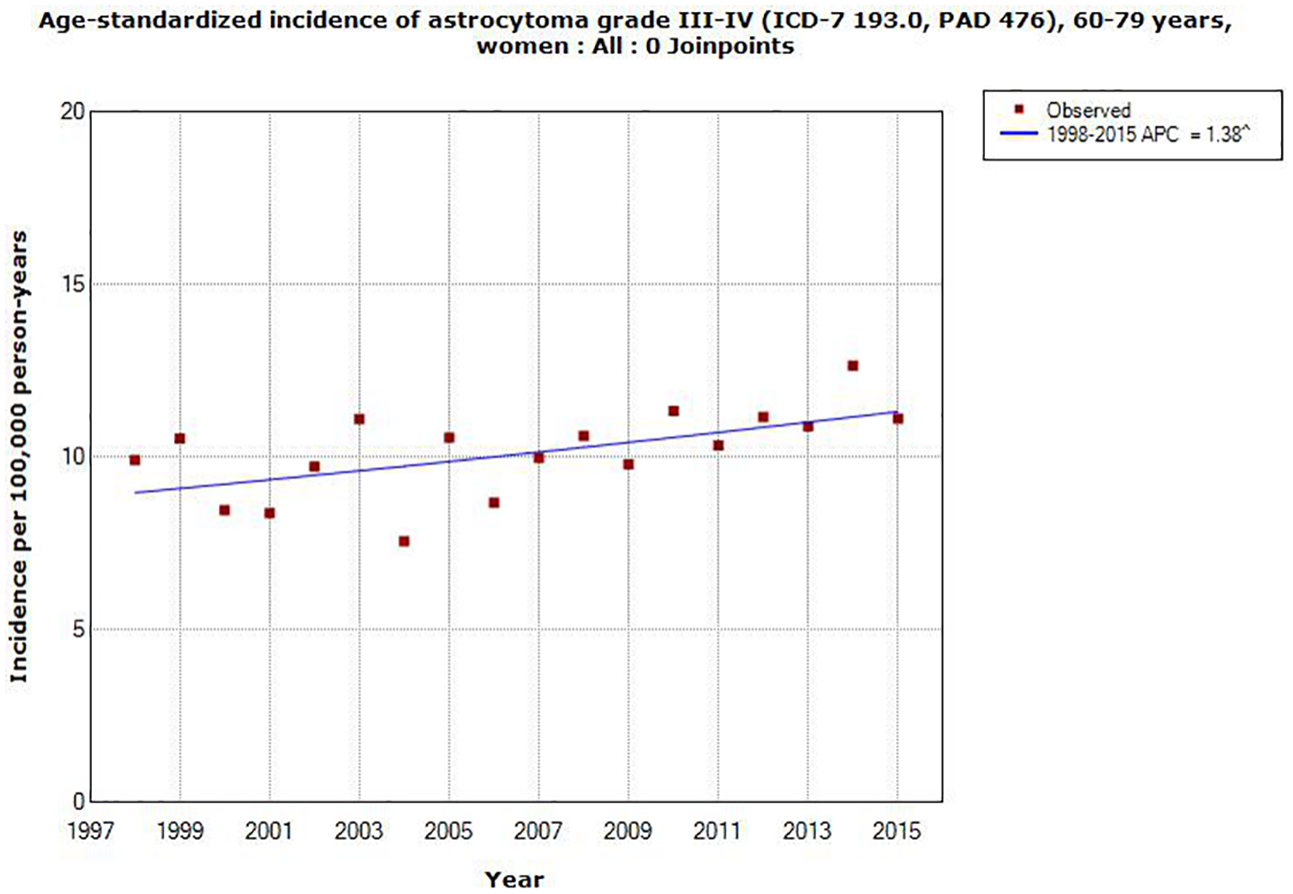
Joinpoint regression analysis of age-standardized incidence rates per 100,000 in women aged 60–79 years with astrocytoma grade III or IV in the Swedish Cancer Register during 1998–2015. (http://www.socialstyrelsen.se/statistik/statistikdatabas/cancer).

AAPC increased in all ages in men for astrocytoma grade III, IV, +0.81, 95% CI -0.002, +1.62%, Table 2. The corresponding result in women was +0.16, 95% CI -0.68, +1.01%. In different age groups statistically significant increase of AAPC was found in both men and women in the age group 60–79 years, AAPC +1.68, 95% CI +0.39, +2.99% and AAPC +1.38, 95% CI +0.32, +2.45%, respectively, see Figs 5 and 6. No joinpoint was detected in any age group, Table 2.

### Wireless phones

Table 3 gives results for use of mobile phones in total and also in different age groups at time of diagnosis. Overall statistically significant increased risk was found for ipsilateral use, OR = 1.8, 95% CI = 1.4–2.2 with highest risk in the age group 18–39 years, OR = 2.2, 95% CI = 1.2–3.8. No statistically significant increased risk was found for contralateral use.

**Table 3:** Odds ratio (OR) and 95% confidence interval (CI) for glioma (n = 1,380) for use of mobile phone (total, ipsilateral, and contralateral exposure), total and in different age groups (age at diagnosis). Number of exposed cases (Ca) and controls (Co) are given. Adjustment was made for age at diagnoses, SEI-code (socio-economic index; blue-collar worker, white-collar worker, self-employed, unemployed), and year for diagnosis. Overall results (all ages) have previously been published [12].

	Mobile phone, all			Ipsilateral			Contralateral		
	Ca/Co	OR	95% CI	Ca/Co	OR	95% CI	Ca/Co	OR	95% CI
All ages	945/2,148	1.3	1.1–1.6	592/920	1.8	1.4–2.2	316/729	1.1	0.8–1.4
-18-39 years	223/361	1.6	1.02–2.6	139/148	2.2	1.2–3.8	69/116	1.0	0.5–2.0
-40-59 years	446/1,073	1.4	1.03–1.8	282/468	1.8	1.3–2.5	151/361	1.2	0.8–1.7
-60-80 years	276/714	1.2	0.9–1.6	171/304	1.6	1.1–2.3	96/252	1.0	0.7–1.6

Table 4 displays the same results for use of cordless phone. The results were similar as for mobile phone use with overall statistically significant increased risk for ipsilateral use, OR = 1.7, 95% CI 1.3–2.1 and highest risk in the age group 18–39 years at diagnosis of brain tumor, OR = 2.4, 95% CI = 1.3–4.5. No statistically significant increased risk was found for contralateral use.

**Table 4:** Odds ratio (OR) and 95% confidence interval (CI) for glioma (n = 1,380) for use of cordless phone (total, ipsilateral, and contralateral exposure), total and in different age groups (age at diagnosis). Number of exposed cases (Ca) and controls (Co) are given. Adjustment was made for age at diagnoses, SEI-code (socio-economic index; blue-collar worker, white-collar worker, self-employed, unemployed), and year for diagnosis. Overall results (all ages) have previously been published [12].

	Cordless phone, all			Ipsilateral			Contralateral		
	Ca/Co	OR	95% CI	Ca/Co	OR	95% CI	Ca/Co	OR	95% CI
All ages	752/1,724	1.4	1.1–1.7	461/766	1.7	1.3–2.1	259/565	1.2	0.9–1.6
-18-39 years	189/303	1.6	1.004–2.6	118/125	2.4	1.3–4.5	58/98	1.0	0.5–2.1
-40-59 years	337/870	1.3	0.97–1.8	200/404	1.4	0.98–2.1	127/279	1.3	0.9–2.0
-60-80 years	226/551	1.5	1.1–2.0	143/237	1.9	1.3–2.8	74/188	1.3	0.8–2.1

## Discussion

All register data in this study were based on official statistics in Sweden without personal id such as name and id number. Only year of diagnosis, type or disease, gender and age at diagnosis were available. For IPR rates were calculated per 100,000, age-standardized incidence is not available in IPR in contrast to the Swedish Cancer Register. It should be noted that the patients are calculated only once each year in these registers. Thus multiple admissions are not included. A person might be included the next year, but it is unlikely that it explains the increasing rates, especially with the joinpoints for D43. Furthermore patients with astrocytoma grade IV (glioblastoma multiforme) have a short survival, usually less than one year.

This study confirmed our previous finding from 2015 of increasing rate of tumors of unknown type in the central nervous system (D43) in the Swedish Inpatient Register (IPR). The follow up period was now two more years, 1998–2015. Similarly as before we found a joinpoint for all ages, both genders combined, in 2007 with high APC during 2007–2015. Regarding different age groups at diagnosis AAPC increased statistically significant in the age groups 20–39, 40–59 and 60–79 years. AAPC was highest for persons aged 20–39 years.

In men and women separately, similar results were found with high AAPC in the age group 20–39 years in IPR. Somewhat higher AAPC was found in women 80+ years, although not statistically significant. No joinpoint was detected in different age groups of men. Women aged 60–79 years had one joinpoint in 2005 and aged 80+ two joinpoints 2001, and 2009. APC increased statistically significant in the latest time period in both age groups.

The age-standardized incidence rate per 100,000 of brain tumors (ICD-7 code 193.0) in the Swedish Cancer Register increased statistically significant in men during 1998–2015. Also in women AAPC increased, although not statistically significant, see Table 2. For astrocytoma grade I, II one joinpoint was found in 2002 in men and APC increased statistically significant during 2002–2015. No joinpoint was found for astrocytoma grade III, IV. The increase of AAPC during 1998–2015 was of borderline significance for these tumor types in men.

In different age groups AAPC for brain tumors increased statistically significant in men aged 80+ in the Cancer Register. These results were based on low numbers and few were of the astrocytoma type. There is no clear explanation for these results that might be chance findings. Also in women AAPC increased in the same age group although not statistically significant.

AAPC increased in all age groups for astrocytoma grade I, II, both in men and women, although not statistically significant, except in women aged 0–19 years with a statistically non-significant decrease. For astrocytoma grade III, IV AAPC increased statistically significant for both genders aged 60–79 years.

The results in the Swedish Cancer Register are in contrast to our previous findings [9]. Thus, for the ICD-7 code 193.0 for the time period 1998–2013 AAPC increased in men +0.06%, 95% CI -0.57, +0.69%, and in women +0.17%, 95% CI -0.60, +0.95%. Higher AAPC was now found in both genders adding two more years (1998–2015). This might reflect exposure to cancer causing agent(s) with reasonable latency period such as wireless phones, but more years are needed to evaluate the time trend.

Clearly this study shows a contrast between the results for D43 in IPR and ICD-7 code 193.0 in the Cancer Register. The rate of brain tumors is increasing in IPR, especially during the latest time period 2007–2015. Highest AAPC was found in the age group 20–39 years for both genders combined in IPR. This is in contrast to the finding in the Cancer Register with highest AAPC in the age group 60–79 years for astrocytoma grade III, IV, although AAPC increased statistically significant also in IPR in that age group.

We discussed at length in our previous article the many shortcomings in the Swedish Cancer Register [9]. We concluded that our results indicated that a large part of brain tumors of unknown type were not reported to the Swedish Cancer Register. In short the diagnosis of nervous system tumors based on autopsy has declined from almost 20% in men and about 15% in women in 1980 to 1% in both genders in 2015. This reflects the general decline of autopsies in Sweden and in fact for all malignancies combined 0% of the diagnoses were based on autopsy in 2015 (http://www.socialstyrelsen.se/SiteCollectionDocuments/2017-1-14-tabeller.xls; Table 30).

Partially this reflects better diagnostic tools such as computer tomography (CT) and magnetic resonance imaging (MRI). In fact even type of brain tumor can be classified using MRI. This would give an increasing number of diagnoses based on clinical investigations and lower numbers based on cytology or histopathology in the Cancer Register. Reports to the Cancer Register can be based on clinical examinations. Brain tumor diagnosis was based on cytology/histopathology in 83% for men and in 87% for women in 1980. This frequency increased to about 90% in both genders in early 1990’s and was 90% in men and 88% in women in 2015. Brain tumors of unknown type represent cases without morphological diagnosis. Thus if they were fully reported to the Cancer Register the number of diagnoses based on cytology/histology would have decreased in the register. Now the opposite was found in the Cancer Register.

With introduction of better clinical diagnostic tools such as CT, MRI and positron emission tomography (PET) morphological diagnosis is less needed. This is especially the situation if biopsy or operation is difficult to perform due to tumor location, age and medical condition of the patient, or treatment is judged not to be possible.

Previously radionuclide brain scan, angiography and pneumoencephalography were used in the diagnostic procedures of brain tumors. The introduction of CT in the late 1970’s provided possibility to diagnose different types of brain tumors such as astrocytoma, meningioma and metastases [15–18]. In the 1980s MRI yielded an important addition for diagnosis of neurological diseases [19,20]. Advanced MR imaging may even separate atypical and more aggressive tumors [21]. The addition of PET-CT provided an independent measure for diagnosis and aggressiveness of the brain tumor [22, 23]. Over the years PET has been increasingly used for management of brain tumors for e.g., diagnosis, grade of malignancy, and operability [24, 25].

The increasing use of non-invasive techniques for diagnosis of brain tumors may explain the differences of the rates in IPR and the Swedish Cancer Register. Thus, we postulate that a number of diagnoses based on these imaging techniques but without cytology/histology are not reported to the Cancer Register but appear in IPR coded as brain tumor of unknown type. It is unlikely that they represent metastases from e.g. breast cancer or melanoma, since they are coded code with the primary disease as main diagnosis. PET may identify metastases [15, 26,27] and may even be used for grading of glioma [28].

A quality control in 2009 of patients dying in cancer showed that 12.5% were never reported to the Swedish Cancer Register [29]. Radiology based diagnosis without a biopsy was the most common diagnostic procedure among these patients. Among pancreatic and biliary tract cancer only 44% and 37%, respectively, were reported to the Swedish Cancer Register during 2005–2009 [30].

We analyzed glioma risk for use of wireless phones at the age of diagnosis. Ipsilateral use yielded increased risk for both mobile and cordless phones in all age groups with highest OR in those aged 18–39 years. Interestingly in IPR ICD-10 code D43 showed highest rate among subjects ages 20–39 years, both genders combined, AAPC +2.71%, 95% CI 1.08, +4.37%. In a previous publication we presented results for age for first use of mobile or cordless phone [12]. Highest risk was found in those persons that started use of the wireless phone before the age of 20. This may be in agreement with the present results in IPR for the age group 20–39 years taking a reasonable latency period. It should be noted that in our case-control study the patients with astrocytoma grade IV and with first use of mobile or cordless phone before the age of 20 had decreased survival with higher hazard ratio than in older age groups [31]. The brain is under development until the age of about 20 years and is more vulnerable than among adults [32].

In summary this register based study showed increasing rates of tumors of unknown type in CNS (D43) with higher rate during 2007–2015. AAPS increased especially in the age group 20–39 years at diagnosis. This may be explained by higher risk for brain tumor in subjects with first use of a wireless phone before the age of 20 years taking a reasonable latency period. Our results indicate underreporting of brain tumor cases in the Cancer Register which undermines a valid evaluation of trends. Incidence trends based on the Cancer Register should be used with caution. However, in spite of the underreporting the age-standardized incidence rate of brain tumors, ICD-7 code 193.0, increased during 1998–2015 in both genders, statistically significant in men.
